# Validation of the Italian version of the Non-Communicating Children's Pain Checklist-Postoperative Version

**DOI:** 10.1186/s13052-017-0388-2

**Published:** 2017-08-22

**Authors:** C. Zanchi, M. Massaro, G. Ferrara, M. Montico, F. D’Osualdo, R. Rutigliano, A. Taddio, L. Vecchi Brumatti, G. Cozzi, E. Barbi

**Affiliations:** 1 Institute for Maternal and Child Health IRCCS “Burlo Garofolo” Trieste, Trieste, Italy; 2 University of Trieste, Trieste, Italy; 3 Institute of Physiatrics and Rehabilitation Gervasutta, Udine, Italy; 4 Pediatric Clinic, Insitute for Maternal and Child Health IRCCS Burlo Garofolo, via dell’Istria 65/1, Trieste, Italy

**Keywords:** Child, Pain, Intellectual disability, Pain assessment

## Abstract

**Background:**

This study evaluated the validity and reliability of the Italian version of the Non-Communicating Children’s Pain Checklist-Postoperative version (I-NCCPC-PV).

**Methods:**

The original NCCPC-PV version was translated into Italian following the guidelines for “the translation, adaptation, and validation of instruments or scales for cross-cultural healthcare research”. We tested the Italian NCCPC-PV version (I-NCCPC-PV) in 40 children (3–18 years of age) with severe to profound Intellectual Disability and no verbal communication. Each child’s behavior was observed by a parent or caregiver and by an external observer in a quiet situation and a painful one. They independently assessed the child’s level of pain using the translated Italian version of the NCCPCPV (I-NCCPC-PV).

**Results:**

The results from 80 assessments showed that children’s behavioral signs differed significantly between painful and calm situations (*p* < 0.001). The inter-rater reliability was poor in a quiet condition (ICC 0.62) and fair in a painful situation (ICC 0.77). The inter-rater agreement was good in both calm and painful conditions (72.50% and 77.50% respectively).

**Conclusion:**

The Italian version of the NCCPC-PV (I-NCCPC-PV) can be used for pain assessment in children with Intellectual Disability who lack verbal communication.

## Background

Pediatric pain management is an important and challenging area of research, especially in children with Intellectual Disability (ID). Patients with ID experience pain more frequently than healthy children: they are at a high risk of chronic conditions and associated diseases which could evoke pain: muscular contractures, chronic constipation, gastro-esophageal reflux, hip-luxation, bone fractures and tooth decay. Moreover, they frequently need invasive diagnostic and therapeutic procedures which can lead to stressful and painful situations, such as botulinum toxin injection, gastrointestinal endoscopy, stomatological treatment, blood sampling and surgical procedures [1, 2]. Pain has a strong negative impact on the quality of life of these children and their families, and it interferes with their ability to perform established skills. Chronic pain has also been associated with sleep disturbances, increased fatigue, depression and decreased physical functioning [3–6]. A study that described the pattern of these children’s typical pain on a daily basis revealed that 35 to 52% of children with moderate to profound ID felt pain each week, and the average time they spent in pain was 9–10 h per week. Children with the poorest abilities resulted in experiencing the greatest pain [3, 7]. Nevertheless, their pain often remains unrecognized and untreated, due to their limited capacities to self-report pain [1].

In the last 10 years, several observational pain assessment tools were developed, mainly based on the observation of physiological and behavioral indicators of pain; unfortunately, these are not routinely used in clinical settings. We recently conducted a telephone interview contacting 56 pediatric wards in three regions of North-Eastern Italy and only 1 center out of 56 reported to use a specific tool for pain assessment in children with CI [8].

The lack of a valid and reliable Italian pain assessment tool makes it difficult for health professionals to evaluate a child’s pain and to provide effective pain treatment, and this results in poor pain management. Recent reports proved that children with ID undergoing surgery receive less opioid infusion in the perioperative period than children without ID [9–11].

The Non-Communicating Children’s Pain Checklist-Postoperative version (NCCPC-PV) is a pain assessment tool specifically designed for children between 3 and 18 years of age with ID, which has been developed from semi-structured interviews with parents of children with severe neurological impairment. It is proved to be a valid and reliable instrument to evaluate pain even when it is used by adults who are not familiar with the patients, and it is easy to perform also in a clinical setting [2, 7, 12, 13]. An Italian version of the NCCPC-PV has not been validated yet.

The aim of our study was to develop and to test the validity and reliability of an Italian version of NCCPC-PV in children with ID, to provide Italian health professionals with a tool to better understand and manage these children’s pain.

## Methods

### Procedures and instruments

We translated the NCCPC-PV following the guidelines for “the translation, adaptation, and validation of instruments or scales for cross-cultural healthcare research” [14]. These consist of seven consecutive steps which represent the framework of the translation process.

**Fig. 1:**
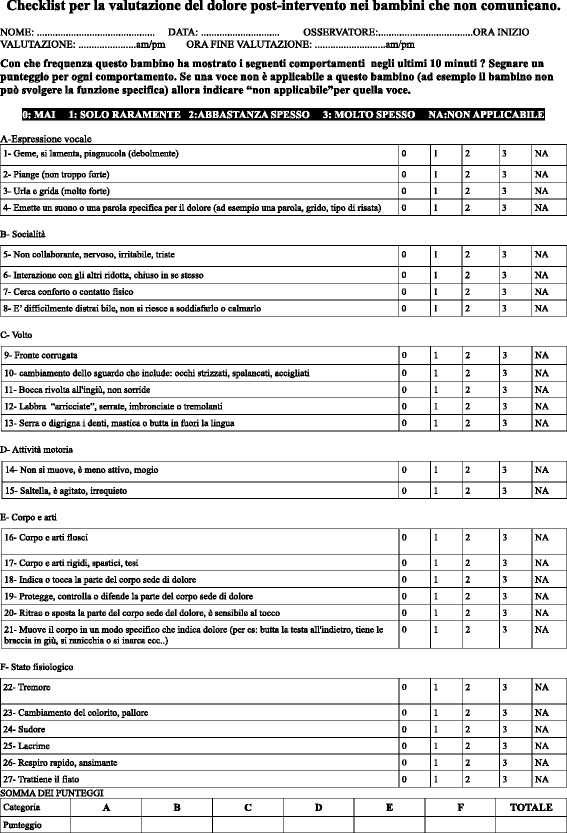
Italian version of NCCPC-PV

The instrument was initially translated from English into Italian (I) by two independent translators, thus producing two Italian versions of the NCCPC-PV (I1 and I2). The former translator was knowledgeable about health terminology while the latter was knowledgeable about cultural and linguistic nuances of the target language. In the second step a third bilingual, independent translator compared the I1 and I2 with the original version of the instrument, to resolve ambiguities and discrepancies, and generated a third version of the scale in Italian (preliminary Italian version PI). In the third step, the PI was translated back to English by two other independent translators, both of them being English native speakers and blind to the original version of the NCCPC-PV. They created two different back-translations of the NCCPC-PV (BT1 and BT2). In step four, a multidisciplinary committee compared the two back-translations (BT1 and BT2) and the original tool. Any ambiguities and discrepancies regarding cultural meaning and colloquialisms or idioms in words and sentences of the items appearing between BT1, BT2 and the original instrument were discussed and resolved through consensus among the committee members to drive the pre-final Italian version of NCCPC-PV (P-FI-NCCPC-PV). In the last step, the P-FI-NCCPC-PV was pilot tested by twenty Italian health professionals who evaluated the instructions, items and response format clarity. Finally, the P-FI-NCCPC-PV was approved jointly as the final Italian version of the NCCPC-PV (I-NCCPC-PV). This scale consists of 27 items divided into six categories: sound, social, facial, activity, body/limb and physiological signs (see Fig. 1). The observers establish a score for each item on a five-step ordinal scale, according to the frequency of its occurrence: 0 = not at all, 1 = just a little, 2 = fairly often, 3 = very often, NA = not applicable. The total score (0–81) is obtained from the sum of each item’s score.

### Subjects

This observational study was carried out at the Department of Pediatrics of the Institute for Maternal and Child Health IRCCS Burlo Garofolo, Trieste, and at the Institute of Physiatrics and Rehabilitation Gervasutta, Udine, in Italy, between April 2012 and October 2016.

The study was approved by the ethical committee of IRCCS Burlo Garofolo, and all parents gave their consent to the study. We recruited consecutive patients with severe to profound ID and inability to communicate verbally, aged between three and eighteen, that were exposed to potentially painful situations, such as blood sampling, botulinum toxin or other intramuscular injections, surgical procedures and so on. Each child’s behavior was observed by a parent or caregiver and by an external observer (medical doctor resident in pediatrics or nurse) during 10 min in a calm situation and then, on the same day, during a painful one; the intensity of pain was evaluated using the I-NCCPC-PV scale. The exclusion criteria were limited to the inability of the parent to understand and speak Italian and limited capability to participate because of tough living conditions.

### Statistical analyses

A linear mixed model was used to evaluate if there were differences in the NCCPC-PV scores rated by the caregiver and by the external observer in the two situations (calm and painful). The NCCPC-PV score was considered as the dependent variable, while raters and situations were taken as the independent variables.

The linear mixed model was also used to calculate the inter-rater reliability, measured with the Intraclass Correlation Coefficient (ICC). The values of the ICC range from 0 (no reliability) to 1 (perfect reliability). ICC values of 0.90–0.99 indicate high reliability, 0.80–0.89 good reliability, 0.70–0.79 fair reliability and below 0.70 indicate poor reliability [14].

NCCPC-PV scores were dichotomized to define presence or absence of pain (≤10 no pain or mild pain; >10 moderate to severe pain) and the percentage of the concordant evaluations was calculated [15].

Values of the NCCPC-PV scale and subscales and age are reported as means and standard deviations (sd). Categorical variables such as sex, diagnosis, procedure and presence of pain are presented as absolute frequencies and percentages. A *p* < 0.05 was considered as statistically significant. All the analyses were carried out with Stata 12.1 (StataCorp LLC – Texas, USA).

## Results

Forty caregivers (38 parents and two private nurses) and four external observers (two medical doctors residents in pediatrics and two nurses) were involved in the assessment of 40 children (14 girls and 26 boys). The children were of 3–18 years of age (median 9, 5 years), according to the original validation study of NCCPC-PV [12]. Twenty had been diagnosed with cerebral palsy, eight children had different specified complex syndromes (2 Aicardi, 1 DiGeorge, 1 CHARGE, 1 Prader Willy, 1 chromosomal deletion, 1 unknown syndrome), five had epileptic encephalopathy, five had delayed psychomotor development of unknown origin, and two children had been diagnosed within the autistic spectrum. All the children were incapable of verbal communication due to severe to profound ID. Table 1 shows clinical and demographical characteristics of the patients.

**Table 1:** Clinical and demographical characteristics of the enrolled patients

	Frequency	Percentage %
Sex		
F	14	35
Diagnosis		
Cerebral Palsy	20	50
Severe Cognitive Impairment	5	12.5
Epileptic Encephalopathy	5	12.5
Syndromes ^a^	7	17.5
Autism	2	5
Chromosomal deletion	1	2.5
Age		
Median (years)	9.5	Range 3–18
Mean (sd)	7.6	Sd 4.6

^a^ 2 cases of Aicardi Syndrome, 1 case of DiGeorge (22q11 deletion), Prader-Willy, Rett, CHARGE, NDD Syndromes

The painful trigger was venipuncture for thirty children, the injection of Botulin Toxin for five children, surgery for scoliosis correction for four children, the placement of a Nasogastric tube for one child (Table 2).

**Table 2:** Painful Situations

Painful situation	N	%
Venipuncture	30	75
Botulin Toxin injection	5	12.5
Post-Surgery^*^	4	10
Nasogastric tube placement	1	2.5
Total	40	100

* 2 fundoplicatio sec Nissen, 1 orchidopexy, 1 scoliosis

**Fig. 2:**
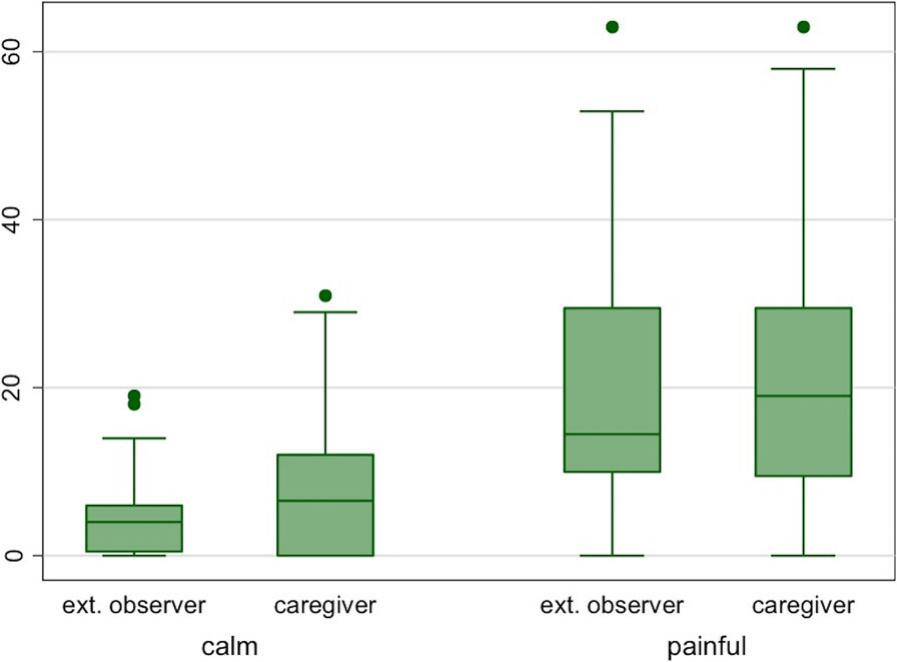
total values of NCCPC-PV in calm and painful situations as rated by the caregiver and the external observer

Figure 2 shows the values of NCCPC-PV in calm and painful situations as rated by the caregiver and the external observer. The values rated by the caregiver are higher than those reported by the external observer in the calm situation (*mean difference* = 3.8, univariate *p* = 0.013), while values were similar in the painful situation (mean difference 0.78, univariate *p* = 0.822). There is no evidence of an interaction between situation and rater. The value of the test significantly changes between calm and painful situation, independently from the rater (adj mean difference14.6, *p* < 0.001).

The inter-rater reliability, measured with ICC, was poor in the calm situation (0.62) and fair in the painful one (0.77). When the values of the test were dichotomized to define presence or absence of pain (<=10 no pain or mild pain; >10 moderate to severe pain), and inter-rater agreement was calculated, it resulted good in both calm and painful situations (72.50% and 77.50% respectively) and also in this case was higher in the painful situation (see Tables 3 and 4).

**Table 3:** Mean and sd of test values, frequencies of raters reporting no pain or mild pain in calm situation and frequencies of raters reporting moderate to severe pain in painful situation

	N	Calm situation		Painful situation	
		Mean (sd)	^a^NCCPC-PV < =10 n (%)	Mean (sd)	^b^NCCPV-PV > 10 n (%)
External observer	40	4.6 (4.7)	36 (90.0%)	20.7 (15.4)	30 (75.0%)
Caregiver	40	8.4 (8.7)	29 (72.5%)	21.5 (15.8)	29 (72.5%)

^a^NCCPV-PC < =10 no pain or mild pain;

^b^NCCPV-PC >10 moderate to severe pain

**Table 4:** Mean and sd of test subscales

	Calm situation		Painful situation	
	Mean	sd	Mean	sd
External observer				
A) VOCAL	0.5	0.9	2.7	2.9
B) SOCIAL	1.1	1.4	4.2	3.2
C) FACIAL	1.4	1.9	5.5	4.2
D) ACTIVITY	0.5	0.8	1.3	1.1
E) BODY AND LIMBS	0.9	1.0	4.3	3.1
F) PHYSIOLOGICAL	0.3	1.2	2.9	3.6
Caregiver				
A) VOCAL	0.9	1.4	2.7	3.3
B) SOCIAL	2.3	2.5	4.5	3.5
C) FACIAL	1.5	1.8	5.1	3.8
D) ACTIVITY	0.7	1.1	1.5	1.3
E) BODY AND LIMBS	2.2	2.7	4.7	3.8
F) PHYSIOLOGICAL	0.9	1.6	3.0	3.0

## Discussion

In this study, we created the Italian version of NCCPC-PV, by using the international translation guidelines, and we tested its reliability in a group of 40 patients with ID. The I-NCCPC-PV total scores resulted significantly higher during a painful situation than during a calm one both for caregivers (21,5 ± 15,8 versus 8,4 ± 8,7) and external observer (20,7 ± 15,4 versus 4,6 ± 4,7), showing that the Italian version of the scale can detect the differences between individual baseline conditions and situations of pain in children with severe to profound ID. The analyses showed a good agreement between the different raters in defining the presence or absence of pain both in calm and painful situations, although the agreement was always better in the painful situations. These results are similar to those reported by Johansson et al. [16] for the validity and reliability of a Swedish version of NCCPC-PV and by Zabalia et al. in the French validation of NCCPC-PV [17].

In this study, in agreement with previous literature [16], I-NCCPC-PV total scores rated by the caregiver were higher than those reported by the external observer both in the calm and painful situation, confirming that it is difficult to rate pain in others by observation and that healthcare personnel (residents and nurses in our study) tend to underestimate pain in children [18]. Hunt et al. [19] have proved that “knowledge of the child” might affect pain assessment.

In our experience, the consistency of evaluation between different observers was poor in a calm situation and fair in a painful situation, with values of the ICC similar to those found in the Swedish experience, indicating limited reliability in a calm situation. When considering the presence or absence of pain (values of <=10 indicating no pain or mild pain; values >10 indicating moderate to severe pain) the agreement between raters was good in both calm and painful situation, proving that this scale is useful to discriminate if a child is in pain or not.

This study has some limitations: the sample of children observed was relatively small, and we considered only one calm and one painful situation for each participant; but our data are comparable to the Swedish version of the scale, which was validated on a sample of 32 children [16]. In our study the painful procedures were performed in two different centers and were not standardized, the external observers were different healthcare professionals, and none was familiar with each child normal behavior.

These points may be seen as limitations, but in our opinion they contribute to the study strength, because the study reflects the normal conditions of pain assessment in a pediatric clinical setting, allowing to confirm the reproducibility of the scale.

These results provide evidence that the I-NCCPC-PV is a useful pain assessment tool for children with severe cognitive impairment. It can be used by healthcare professionals who are not familiar with the patients and could be useful to help clinicians provide a better pain treatment in this subset of particularly difficult patients.

## Conclusions

Observational pain assessment can be difficult: pain can be over or underestimated either by healthcare professionals or by caregivers [20, 21], but it is the only way to recognize and quantify pain in patients with severe CI, and it should be performed with dedicated tools [22].

Translation and validation of these tools in different languages is relevant in clinical practice to provide healthcare professionals and caregivers with an additional instrument to interpret their patient/child behavior and improve pain management. We conclude that the Italian version of NCCPC-PV can discriminate between calm and painful situations in non verbal children with ID based on a brief observation. We hope that the development of a validated Italian version can improve the care of patients with severe to profound ID in our country.

## Abbreviations

ICC: Intraclass Correlation Coefficient
ID: Intellectual Disability
I-NCCPC-PV: Italian non-communicating children’s pain checklist-postoperative version
NCCPC-PV: Non-communicating children’s pain checklist-postoperative version
P- FI-NCCPC-PV: Pre-final Italian version of non-communicating children’s pain checklist-postoperative version
